# Potential Similarity of Serum Cholesterol Multivariate Coefficients in the Prediction of Coronary Heart Disease Across Populations: A Review From the Seven Countries Study

**DOI:** 10.31083/RCM46488

**Published:** 2026-02-26

**Authors:** Alessandro Menotti, Paolo Emilio Puddu

**Affiliations:** ^1^Association for Cardiac Research, 00182 Rome, Italy; ^2^EA 4650, Signalisation, Électrophysiologie et Imagerie des Lésions D’Ischémie Reperfusion Myocardique, Normandie Université, UNICAEN, 14000 Caen, France

**Keywords:** cholesterol, coefficients non-heterogeneity, cross-population studies, Seven Countries Study, CHD mortality, absolute versus relative risks

## Abstract

This study aimed to review the unique contribution of the Seven Countries Study (SCS) of cardiovascular diseases to the possible non-heterogeneity in multivariate coefficients of serum cholesterol in predicting coronary heart disease (CHD) mortality across different populations. This study reviewed five published analyses from the SCS, which together encompassed 16 cohorts of middle-aged men from eight nations across seven countries in the USA, northern and southern Europe, and Japan. In total, these analyses included 12,763 participants and follow-up periods ranging from 25 to 60 years after the baseline examination. Serum cholesterol was measured using uniform, standardized procedures that differed from those reported in the literature. Marked differences in mortality rates were observed, with higher rates in the USA and northern Europe and lower rates in the other regions. A systematic comparison of serum cholesterol coefficients did not reveal significant heterogeneity across cohort combinations or follow-up durations of 25, 40, 50, and 60 years for CHD mortality. In all cases, coefficients were adjusted for three additional risk factors: age, cigarette smoking, and systolic blood pressure. Variations in CHD mortality rates across populations were explained by differences in serum cholesterol levels. In contrast, the magnitudes of serum cholesterol coefficients were relatively similar across groups, although not necessarily homogeneous. These findings support the idea that predictive models of CHD mortality developed for a specific population can also be applied to other populations, since the expectation, at least for serum cholesterol, is to obtain similar relative risk estimates, not absolute risk, which aligns with the limited evidence that is available in the current literature.

## 1. Introduction

There is consensus on the role of total serum cholesterol levels as a major and specific predictive risk factor for coronary heart disease (CHD), with applicability to both individuals and populations [1]. Here, we report data on the possible similarity of serum cholesterol multivariate coefficients in predicting CHD events across populations, which could facilitate cross-population application of risk functions. The immediate consequence is the identification of potential common mathematical relationships linking risk factor levels to overall CHD risk.

This review aimed to summarize and comment on published data collected over 60 years in a series of papers from the Seven Countries Study (SCS) of cardiovascular diseases, and to compare the multivariate coefficients for serum cholesterol in predicting CHD mortality across different populations. This analysis pertains only to the SCS, a unique multicenter study conducted across several cohorts using the same methodology.

## 2. Overview of the Seven Countries Study

The SCS originated in 1958, enrolling 16 cohorts of middle-aged men (aged 40–59 years) with contrasting eating habits for a total of 12,763 participants, in seven countries, *i*.*e*., the USA, Finland, the Netherlands, Italy, the former Yugoslavia republics (Croatia and Serbia), Greece, and Japan. There were 10 rural communities, one fishing community, four occupational groups, and one demographic sample. CHD prevalence, incidence, and mortality, as well as rates of other cardiovascular events, were compared over a long follow-up period. The entry examination included family and social data, lifestyle behaviors, including smoking and working habits, a series of anthropometric measurements, a few biochemical and biophysical measurements, diagnoses of major diseases obtained by a complete medical examination, recording of a resting and post-exercise electrocardiogram, and resting spirometry measurements. A complex dietary survey was conducted using subsamples from each cohort collected at the homes of the participants, thereby enabling the recording of many food groups and the chemical measurement of basic nutrients in portions of the consumed food. Follow-up procedures comprised quinquennial field re-examinations for up to 40 years—although not for all cohorts—along with the collection and coding of mortality data up to 60 years of follow-up in 10 practically extinct cohorts, and shorter follow-up periods in the remaining six cohorts. Mortality data were coded according to defined rules using the 8th Revision of the World Health Organization (WHO) International Classification of Diseases [2]. Major information on the study can be found in three monographs [3, 4, 5], while other important findings were published later.

The SCS provides a unique opportunity to investigate issues using data from 16 cohorts of men, all in the same age range, followed up for decades, with consistent chemical methods for serum cholesterol measurements, uniform follow-up periods, and identical objective diagnostic criteria for causes of death. This was complemented by large differences in CHD mortality across the 16 cohorts, which, depending on the type of cohort grouping and the length of follow-up, ranged from 2.1 to 7.8 times, as reported in Tables 1 and 2 (Ref. [6, 7, 8, 9, 10]).

**Table 1.** **SCS contribution for 25 years of follow-up (two papers)**.

PAPER 1: 1995 JAMA [6]	
Population	16 cohorts across eight nations with 12,763 men aged 40–59
	Compacted in six geographical areas: USA, Northern Europe, Mediterranean South Europe, Inland South Europe, Serbia, Japan
Endpoint and follow-up	CHD mortality over a 25-year follow-up
Models	Cox models with CHD mortality as the endpoint, versus serum cholesterol plus age, systolic blood pressure, and cigarette smoking as confounding covariates
Relative risk for 20 mg/dL of cholesterol difference	Levels are very similar across areas, except perhaps those of Japan: range 0.96 to 1.15 (including Japan), 1.10 to 1.15 (excluding Japan)
	Estimates increased by 40% after adjustment for regression dilution bias
Curves of baseline cholesterol levels versus CHD mortality	Almost parallel but at different levels
Conclusions	Coefficients of serum cholesterol are not different across various geographical areas, but the heterogeneity test was unavailable
PAPER 2: 1996 Journal of Cardiovascular Risk [7]	
Population	12,763 men, aged 40–59 years in 16 cohorts across 8 nations: USA, Finland, the Netherlands, Italy, Croatia, Serbia, Greece, Japan
Endpoint and follow-up	CHD mortality over a 25-year follow-up
Models	Cox models with cholesterol plus age, systolic blood pressure, and cigarette smoking as confounding covariates
Test of serum cholesterol coefficients heterogeneity across nations	*p* = 0.5492
	*p* = 0.2323 including interaction cholesterol/nation
Comparison of cholesterol coefficients in all pairs of nations	Out of 28 comparisons, 23 were not significant: after adjustment for multiple correlations, 27 comparisons were not significant
Explanations of correlations R^2^	Coefficients versus CHD death rates: R^2^ = 0.09
	Mean cholesterol versus CHD death rates: R^2^ = 0.80
Conclusions	Multivariate coefficients of cholesterol do not differ across nations
	Multivariate coefficients of cholesterol do not relate to national death rates

Abbreviation: SCS, Seven Countries Study; CHD, coronary heart disease.

**Table 2.** **SCS contribution for 40, 50, and 60 years of follow-up (three papers)**.

PAPER 3: 2008 European Journal of Cardiovascular Prevention and Rehabilitation [8]	
Populations	10,157 men aged 40–59 years across seven nations: USA, Finland, the Netherlands, Italy, Serbia, Greece, Japan
Endpoint and follow-up	CHD mortality over a 40-year follow-up
Models	Cox models with CHD mortality as endpoint, versus serum cholesterol plus age, systolic blood pressure, and cigarette smoking as confounding covariates
Special measurement of cholesterol for analysis	Use of baseline, then up to 3 measurements in 10 years, and up to 6 measurements in 35 years
Test of serum cholesterol coefficients heterogeneity across nations	Baseline: *p* = 0.2691
	Up to 3 measurements: *p* = 0.5802
	Up to 6 measurements: *p* = 0.8168
	Time dependent up to 3 measurements: *p* = 0.1220
	Time dependent up to 6 measurements: *p* = 0.2932
Curves of CHD mortality as a function of baseline cholesterol levels	Practically parallel, although located at different levels
Comments	The use of up to 3 or 6 measurements of cholesterol partially improves the predictive value of serum cholesterol
Conclusions	Serum cholesterol coefficients do not differ across various nations, even when different analytical approaches are applied
PAPER 4: 2018 Acta Cardiologica [9]	
Population	11 cohorts across seven nations with 10,368 men aged 40–59 years, compacted into five geographical areas: USA, Northern Europe (Finland plus the Netherlands), Mediterranean (Italy plus Greece), Serbia, Japan
Endpoint and follow-up	CHD mortality over a 50-year follow-up (45 years for Serbia)
Models	Cox models with CHD mortality as the endpoint, versus serum cholesterol plus age, systolic blood pressure, and cigarette smoking as confounding covariates
Test of serum cholesterol coefficients heterogeneity across geographical areas	*p* = 0.6235 for 4 areas with 50 years of follow-up
	*p* = 0.8817 for 5 areas with 50 or 45 years of follow-up
Test of cholesterol coefficients on all possible pairs of areas	*p* of differences ranging from 0.2250 to 0.8352 in 10 comparisons
Conclusions	Coefficients of serum cholesterol are not different across various geographical areas despite the follow-up close to extinction
PAPER 5: 2022 Journal of Cardiovascular Medicine (Hagerstown, Md.) [10]	
Population	Seven cohorts from four European nations, reduced to two areas of north versus south Europe (Finland plus the Netherlands) versus (Italy plus Greece): n = 5152
Endpoint and follow-up	CHD mortality over a 60-year follow-up in extinct cohorts
Models	Cox models with CHD mortality as the endpoint, versus serum cholesterol plus 13 cardiovascular risk factors as possible confounders
Test of serum cholesterol coefficients heterogeneity across geographical areas	Coefficients: northern Europe = 0.0034, southern Europe = 0.0051; *p*-value of difference = 0.8829 (not significant)
Conclusions	Coefficients of serum cholesterol do not differ between the two areas

The data reported here deal with serum cholesterol levels measured centrally using the same analytical technique [11] and CHD death rates. We used the test of heterogeneity versus homogeneity proposed by Dyer in 1986 [12] to evaluate possible similarities among cholesterol coefficients; high *p*-values (>0.05) indicate the absence of heterogeneity. The analysis examined five papers largely dedicated to the problem [6, 7, 8, 9, 10]; for each, the study reports the cohorts or nations involved, the endpoint, the follow-up duration, the structure of the predictive model, the test for heterogeneity, and, occasionally, other details. Nevertheless, it is important to recall that the SCS provided evidence of a direct relationship between serum cholesterol and CHD, both at the ecological and individual levels, as reported in the main monographs [3, 4, 5] and in more recent papers [8, 9, 10, 13].

## 3. Evidence on Serum Cholesterol and CHD Mortality

Tables 1 and 2 summarize the outcomes of the published analyses related to the different follow-up periods of 25 to 60 years [6, 7, 8, 9, 10]. The terminology is clarified as follows: (a) cohorts (16 in total) are samples of subjects representing the basic units for this type of analysis; their names derive from geographical locations and/or occupational characteristics; (b) countries (7 in total) are the “political” recognized entities at the beginning of the study; (c) nations (8 in total) are the operational definitions used when the five cohorts located in former Yugoslavia were segregated into the two internal federated republics (Croatia and Serbia), accounting for the differences in their ethnical and cultural background; (d) areas (variable number) are the geographical–cultural entities artificially created by combining cohorts or nations belonging to different countries/nations. In each of the five analyses, the number of the statistical units is provided in “cohorts”, or “nations”, or “areas”. Since this is a review of published data, no additional analysis or results were derived from the original data.

The findings reported in Tables 1 and 2 are self-explanatory when examined carefully and are summarized concisely in words and numbers. The magnitudes of the multivariate serum cholesterol coefficients (in all cases adjusted for 3 additional major risk factors) do not differ across the various population groups defined in each analysis. The main finding is the systematic non-significant outcome from the heterogeneity test (although not computed on the data from paper 1 [6]). Moreover, paper 2 [7], presented in Table 1, showed that the population mean serum cholesterol levels explain 80% of the observed variance in the CHD death rates (significant). In contrast, the magnitudes of the serum cholesterol coefficients explain only 9% of the variance in the CHD death rates (non-significant). This suggests that the population multivariate cholesterol coefficients do not explain the relationship between serum cholesterol and CHD death rates.

The original figures in papers 1 and 3 [6, 8], which reported 25- and 40-year follow-ups, showed parallel trends, suggesting similar relative risks across different mean serum cholesterol levels. However, the curves were located at various levels, indicating that the observed CHD death rates are due to differences in serum cholesterol levels across the populations, adjusted for three other risk factors. The increase in the serum cholesterol hazard ratio following adjustment for regression dilution bias, presented in paper 1 [6], was indirectly confirmed by the higher *p*-values of the homogeneity versus heterogeneity tests described in paper 3 [8], using multiple measurements of serum cholesterol.

Interestingly, similar findings were obtained for follow-ups of different durations, from 25 to 40, 50, and 60 years. We do not claim that the coefficients are homogeneous or equal; however, since the coefficients appear to be non-heterogeneous, a special meaning may be derived: death rate levels among cohorts are not related to the size of cholesterol coefficients.

## 4. Consistency of Multivariate Cholesterol Coefficients Across Populations

The cholesterol multivariate coefficient can be translated into a slope describing the relationship between differences in CHD fatality risk and serum cholesterol levels. We have shown that the magnitudes of these coefficients are rather similar across cohorts, while the coefficients are unrelated to the death risk and the rates of the same cohorts. This also means that the relative risk of an event for a given difference in baseline serum cholesterol is similar in the various populations.

**Fig. 1.**
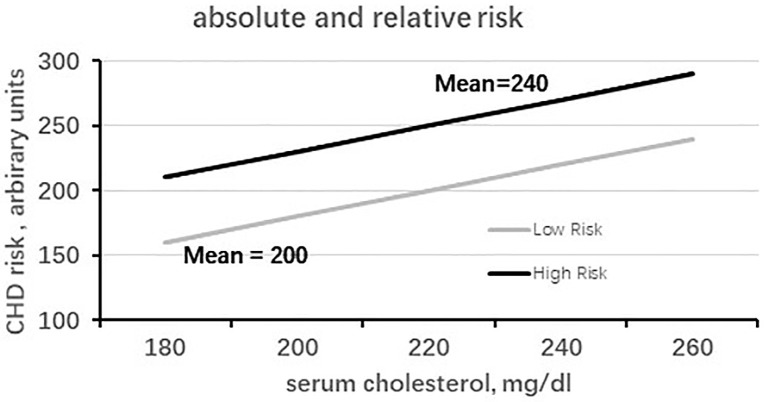
**Simulation of the relationship of high- and low-risk populations for cholesterol versus coronary heart disease (CHD) risk, in terms of absolute and relative risks**. Upper straight line: baseline serum cholesterol of a high-risk population; the slope is derived from the cholesterol coefficient of multivariate analysis versus CHD risk (in mg/dL). Lower straight line: baseline serum cholesterol of a low-risk population; the slope is derived from the cholesterol coefficient of multivariate analysis versus CHD risk (in mg/dL). Ordinate: scale of CHD risk in arbitrary units.

To facilitate understanding of the reported findings, we recall a few notions regarding absolute and relative risks, partly depicted in Fig. 1, in which numerical components are presented in arbitrary units, and the entire system is a simulation. Absolute risk is the probability of an event as a function of different levels of baseline risk factor (serum cholesterol in our case). Fig. 1 shows the two slopes derived from serum cholesterol coefficients in a population with low cholesterol (mean around 200 mg/dL) and another with high cholesterol (mean around 240 mg/dL). The consequential absolute risks differ and roughly correspond to the intercept of the mean serum cholesterol levels and the ordinate, with values of 160 and 270 arbitrary units on the ordinate scale. The two risks are unrelated to the magnitude of the multivariate coefficients because their levels are not heterogeneous. Conversely, relative risk is the ratio of two probabilities resulting from a difference between two chosen levels of serum cholesterol, exhibiting the differential risk between the levels. Fig. 1 shows that the influence of the ratio, for example, of 220/200 mg/dL, will have the same effect on the corresponding levels of the risk in the ordinate, either coming from the high or low risk groups, since the ratio derives from equal slopes of the two groups.

All the concepts illustrated above relate to the long-standing problem of applying a risk function derived in one population to another, particularly when risk functions were available only in a few countries. The use of coefficients from a high-risk population to predict absolute risk in a low-risk population inevitably overestimates risk; conversely, applying a low-risk function to a high-risk population underestimates risk. This abnormal situation led to complex recalibration procedures, which were often necessary in the past when absolute risk functions were not yet available in all countries [14]. Comparatively, relative risk can be easily and accurately estimated when the coefficients for the two populations are equal, or not significantly different, and this holds regardless of whether the estimates are derived from an absolute high- or low-risk population.

In the early phases of cardiovascular epidemiology, there was some interest in explaining population differences in CHD incidence or mortality as a function of major risk factors, with particular emphasis on serum cholesterol, but little interest in details such as comparing multifactor coefficients across major risk factors. For example, this approach included comparing CHD rates among Japanese living in Hawaii versus Japan [15], comparing the Yugoslav cardiovascular study with the Framingham Heart study [16], and comparing the Framingham Heart study with the Honolulu and Puerto Rico populations [17].

Subsequently, more studies adopted a similar approach, as interest shifted to comparing CHD predictive risk functions. A review of 17 population studies showed that the difference in population mean serum cholesterol explained about 80% of the geographical variation of CHD mortality [18], similar to that found in the SCS [7]. Conversely, an ecological analysis of cohorts in the WHO-MONICA project concluded that traditional risk factors explained only a small portion of the variation in CHD mortality rates. This was particularly true for serum cholesterol, which accounted for no more than one-quarter of the variation, and no multivariate comparisons were provided [19].

Early attempts to compare the risk functions of the Pooling Project Research Group and the Framingham Heart Study did not reach clear conclusions [20, 21]. A meta-analysis of 27 population studies found that relative risk estimates for major risk factors differed substantially across cohorts, with *p* for heterogeneity <0.0001 [22], thus excluding a common, unique predictive function. However, in this case, the favorable (standardized) procedures used in the SCS were not met.

An international case–control study performed in 52 countries evaluated the role of traditional risk factors in myocardial infarction cases versus controls, confirming the role of those risk factors [23]. However, these findings should be interpreted with caution, given the case–control design of the study. A review of 15 studies showed a relatively similar impact of major risk factors on CHD events; however, the possibility of applying the risk function from one population to another was excluded [24].

A Framingham CHD predictive function was applied to various population groups in the USA, characterized by different ethnicities and socioeconomic status, and the results were reasonably good for relative risk. However, recalibration was needed to improve prediction of absolute risk due to large differences across groups in the prevalence of certain risk factors [25]. Another US study compared the Framingham risk function with that of a multicenter Chinese investigation [26].

A comparison of 325,000 middle-aged men screened in the US-MRFIT project with 20,000 men in the same age range examined in the Italian Risk Factors and Life Expectancy (RIFLE) project showed substantial similarity in the magnitude of multifactor coefficients for major CHD risk factors estimated by similar multiple logistic functions. This outcome was also true for serum cholesterol; the difference between the two coefficients was not significant, but caution was applied in the interpretation due to many structural differences between the two studies and the difficulty in comparing the constants of the two models [27].

An extensive meta-analysis involving 61 different studies with 900,000 subjects and 34,000 CHD fatal events produced a side-analysis (confined in an Appendix) showing that there was no heterogeneity across the multivariate serum cholesterol coefficients comparing three large geographical groups: European countries (with 43 studies), USA and Australia (with 12 studies), and East Asia (with 5 studies) [28]. However, the comparisons did not involve single cohorts but instead comprised extremely large cohort pools, which probably diluted any possible differences; moreover, the methodologies across the various studies were largely different.

An interesting observation emerged from the three SCS cohorts in Serbia (former Yugoslavia), where large increases in mean serum cholesterol were observed during the first 25 years of follow-up. However, despite this observation, the multivariate coefficients of serum cholesterol did not change [29, 30].

## 5. Clinical and Epidemiological Implications

One of the most widely evaluated risk function models for predicting CHD and CVD is the EURO-SCORE, funded by the European Union, which includes adults from 12 European countries, totaling 205,000 subjects with over 5600 CHD fatal events over 10 years [31]. In that case, two different models were produced, one for high- and the other for low-risk countries. Many investigators have tested the system in independent population groups across several European countries (Austria, Spain, Germany, Belgium, and the Netherlands [32, 33, 34, 35, 36]) and in Australia [37]. In practically all cases, there was an overestimation of absolute risk, necessitating complex recalibration procedures that, in the end, yielded a reasonable estimate.

A number of the above-mentioned reports had a limited objective of establishing the ecological and/or individual relationship between serum cholesterol and CHD events, while disregarding the issue of the homogeneity versus heterogeneity of risk factor coefficients. Other studies have examined the feasibility of applying the risk function of a population to another. Frequently, this operation produced relatively good results for estimating relative risk but not for estimating absolute risk, resulting in large over- or under-estimates of the latter. This was a good basis for hypothesizing that the multivariate coefficients of major risk factors might have similar magnitudes; however, a real effort was seldom made to provide a final numerical demonstration. However, clear difficulties remain in producing a credible, comparable estimate of risk as a function of a risk factor; several conditions are needed to ensure comparability. In particular, the same sex, age range, historical period, the same analytical methodology for cholesterol measurements, the same length of follow-up, and the same diagnostic criteria for coding causes of death. All these preconditions are essential for valid comparisons, and were rarely met in the literature, not even in large meta-analyses. In general, tests of homogeneity versus heterogeneity for statistical comparison were seldom reported. Importantly, the structure and standardizations of all the above-mentioned preconditions were met by the SCS design, thus enabling our dedicated analyses.

Presently, the problems related to the difficulties of using risk functions derived from certain populations for others have largely been overtaken, as many countries, including some developing ones, employ specific risk functions for CHD prediction. Nonetheless, two open questions remain: first, whether the possible homogeneity of multivariate coefficients can be extended to other major risk factors, such as blood pressure and smoking habits; actually, some preliminary tests performed by our research group have appeared promising [9]; second, to explore the possibility that the homogeneity of multivariate coefficients (in larger number than just cholesterol levels) may hide general biological rules connecting risk factors and the associated relationships with the occurrence of CHD and cardiovascular events in general. However, we are currently unaware of any multicenter studies that have addressed these problems.

## 6. Conclusions

This analysis, which combines a series of published SCS data, has shown that the multivariate coefficients of serum cholesterol predicting CHD events across different populations are not distinct and are unrelated to population mean serum cholesterol and to population death rates. These facts support the use of risk functions from one population to estimate relative risk in another, while no assurance can be given for the estimate of absolute risk. Moreover, these findings suggest well-defined mathematical relationships linking variation in serum cholesterol levels to actual risk, provided that precise preconditions are met. However, the final proof currently remains unavailable.
